# Modeling of the Chemical Re-Alkalization of Concrete by Application of Alkaline Mortars

**DOI:** 10.3390/ma19020278

**Published:** 2026-01-09

**Authors:** Clarissa Glawe, Rebecca Achenbach, Michael Raupach

**Affiliations:** Institute of Building Materials Research, RWTH Aachen University, 52062 Aachen, Germany

**Keywords:** carbonation, chemical re-alkalization, corrosion, durability

## Abstract

Since the number of existing steel-reinforced concrete buildings affected by carbonation-induced corrosion is steadily increasing, there is a high demand for durable repair methods. Chemical re-alkalization (CRA) represents one such approach, relying on the transport of alkaline pore solution from a repair mortar into carbonated concrete. With the introduction of clinker-reduced binder systems such as hybrid alkali-activated binders (HAABs), their suitability for CRA and governing material parameters require further clarification. In this study, material-related chemical and structural influences on CRA were investigated using an adapted form of Fick’s second law of diffusion, incorporating a time-dependent attenuation factor, β(t). The CRA progression was evaluated over 28 days, distinguishing between an initial suction phase and a subsequent diffusion phase. The results show that a high initial alkalinity of the mortar pore solution (pH > 14) significantly enhances re-alkalization during the suction phase, reflected by suction factors a > 1. In contrast, progression during the diffusion phase is primarily governed by the potassium concentration gradient at the mortar–concrete interface, while structural parameters such as capillary porosity show no systematic correlation with the deceleration factor b (−0.46 ≤ b ≤ −0.26). The findings indicate that, within the investigated range, mortar pore solution chemistry has a stronger influence on CRA than structural properties, providing guidance for the targeted design of alkaline repair mortars.

## 1. Introduction

The repair of carbonation-induced corrosion in steel-reinforced concrete structures is one of the fundamental tasks that promote the maintenance and rehabilitation of existing buildings. In addition to chloride-induced corrosion, the carbonation of concrete is the most common durability-reducing process in the case of steel-reinforced concrete. There are different repair methods that can prevent carbonation from progressing further in the direction of steel reinforcements and also restore the passivity of reinforcements. Chemical re-alkalization (CRA) is one of these methods and consists of the application of an alkaline repair mortar on carbonated concrete to introduce an alkaline pore solution from the mortar into the concrete to increase the pH of the concrete’s pore solution. CRA represents a repair method aimed at extending the service life of existing steel-reinforced concrete building structures without extensive concrete removal to contribute to resource-efficient and durable rehabilitation measures.

To be used as a repair material for CRA, a mortar must meet various requirements, which are defined in [1,2]. Above all, the mortar must be able to mobilize alkalis for their transport into the concrete. If the carbonation depth is increased to up to 40 mm, which is the maximum carbonation depth to be re-alkalized by CRA according to German guidelines for repair [3], alkalis must be available over a specific period of time to ensure high penetration depths.

In the case of cement-based mortars, the pore solution remains highly alkaline for a long period of time [4,5]. The hydration of OPC forms a pore solution saturated with Ca(OH)_2_, which can typically have a pH of up to 13 [6]. Even if carbonation leads to the binding of Ca(OH)_2_ during the formation of carbonates (CaCO_3_), this can be buffered by its solid form, portlandite, and later by the decalcification of the C-S-H phases [7,8].

The use of clinker-reduced to clinker-free binders leads to a different composition of the pore solution, which means that the suitability of these binders for use in repair mortars cannot always be assumed. However, alkaline activation of these binders can create a high pH value that can be maintained over a longer period of time [9]. Nevertheless, it remains to be clarified whether a high pH value indicates a mortar’s suitability for chemical re-alkalization. Initial studies on the use of cement-reduced and alkali-activated binders are available that confirm their suitability for chemical re-alkalization [10,11]. In these hybrid alkali-activated binders (HAAB), the cement is substituted by metakaolin to 50%, while sufficient strength is ensured by alkaline activation using potassium hydroxide and water glass. Within the scope of this work, CEM I was used as a reference binder with a well-established pore solution chemistry, while partial substitution by metakaolin in the investigated hybrid alkali-activated binders was intentionally applied to systematically modify the binder’s chemistry in order to investigate its influence on the chemical re-alkalization process [4,12,13,14].

To date, investigations on the use of hybrid alkali-activated binders as repair mortars for CRA are limited. Existing studies have primarily focused on the achievable re-alkalization depths under defined laboratory conditions, including variations in carbonation depth, while the influence of environmental conditions and different concrete substrate properties has not yet been systematically addressed. In contrast, when using cement-based mortars, the influence of ambient humidity on CRA has been predominantly investigated [15,16]. Using HAABs for re-alkalization mortars, re-alkalization depths of up to 33 mm can be achieved under specific conditions, such as cyclic watering for concretes with extremely high porosity [11]. Compared to OPC-based mortars, for which a maximum re-alkalization depth of only 14 mm has been demonstrated so far, the use of HAABs allows for a significant increase in the maximum re-alkalization depth [17]. However, since it is currently assumed in a German guideline that non-carbonated concrete can re-alkalize in so-called rear-sided re-alkalization at up to 20 mm, a re-alkalization depth of 15 mm through the mortar would be sufficient, e.g., with a typical concrete cover of 35 mm [1,18]. Rear-sided re-alkalization, however, has not yet been reported. During the re-alkalization process, the re-alkalization rate changes as a function of time, resulting in a non-linear progression of the process. The progression of CRA when using HAABs is primarily characterized by a strong progression during the first 24 h after mortar application, also referred to as the suction phase [10,19]. The suction phase involves a combination of moisture and concentration gradients between mortar and concrete, leading to pronounced transport after mortar application. The transport mechanism during this phase is often referred to piggyback transport, which describes the convective penetration of alkalis dissolved in water in the form of the mortar pore solution [20,21]. Different re-alkalization depths have been observed depending on the applied mortar, suggesting a strong dependence on the mortar used during the suction phase of CRA. Accordingly, a high initial alkalinity in the form of a high pH value is crucial for the course of CRA, but no pH values are yet available for the HAABs used.

Comparing CRA progression when using HAABs with that when using cement-based binders, the latter exhibits a less pronounced suction phase [19]. This can be explained by a slower pH adjustment, which, in the case of HAABs, is immediately present due to the addition of strongly alkaline solutions to the freshly prepared mortar and results in steep concentration gradients between the mortar and concrete shortly after mortar application [22,23]. While such a pronounced suction phase may suggest strong alkali depletion in the mortar, available observations indicate that it does not necessarily result in a stronger deceleration during the subsequent diffusion phase. Instead, the progression of CRA in the later state appears to remain governed by the prevailing concentration gradient at the mortar–concrete interface. In comparison, CRA progression when using cement-based mortars shows a more steady re-alkalization process, which, although slower, also shows increasing re-alkalization depths over longer time periods, which is defined as the diffusion phase [19]. Although Merkel already distinguished between the suction phase and the diffusion phase based on the temporal evolution of the re-alkalization depth, no standardized methodology exists for the distinction of these two phases [19]. Furthermore, no established model or formula is currently available that enables the selection or optimization of repair mortars for CRA based on quantitative differentiation of these phases.

**Figure 1:**
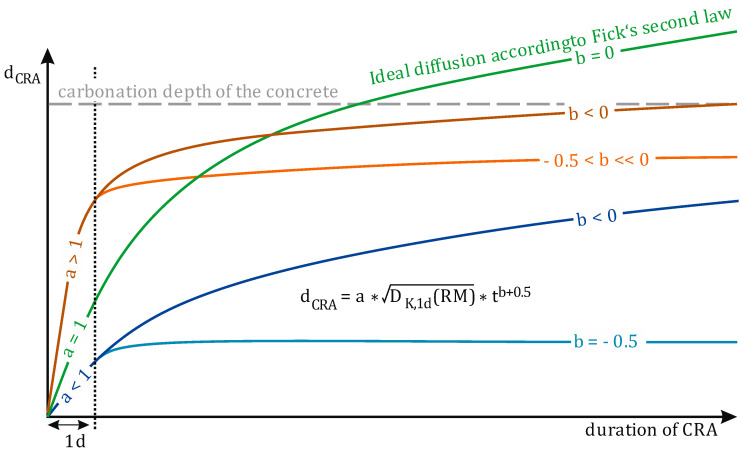
Schematic representation of the adapted diffusion law by Glawe et al. and the impacts of the material-specific factors a and b on the time-dependent evolution of d_CRA_ [10].

As the mortar hardens, not only the pH value but also the structure of the binder is assumed to influence the transport of alkalis from the mortar into the concrete, as well as the structure of the concrete [10,24]. As the re-alkalization period progresses, not only does the availability of alkalis decrease, but the developing microstructure of the mortar also influences the re-alkalization progress. Glawe et al. summarized this as an attenuation factor β(t), which describes the time-dependent systematic deviation of the transport processes during CRA from ideal diffusion [10]. The temporal evolution of the attenuation factor follows the power law β(t) = a * t^b^, which is used to adapt Fick’s second law of diffusion to the CRA. Through the variations in a and b, Figure 1 clearly shows which part of CRA is primarily influenced by each respective factor.

Factor a determines the initial course of CRA within the suction phase. This assumes that the CRA is only considered after one day. Since data on the CRA at a time before one day is generally unavailable, it is not represented in detail by the adapted equation. However, the results of Glawe et al. clearly demonstrate that this simplified assumption does not lead to excessive loss of plausibility regarding the calculated re-alkalization depths at a later time. Since a can also be understood as a suction factor for the diffusion coefficient D_K,1d_(RM) of potassium in the substrate concrete after 1 day of CRA, depending on the applied repair mortar, a value of 1 equals ideal diffusion, while a < 1 results in a comparably lower d_CRA_ and a > 1 in a higher d_CRA_ at the end of the suction phase. In a first assumption based on the investigations of Glawe et al., a is influenced by the applied mortar, as it is proportional to its potassium content [10].

Therefore, factor b defines the extent to which the progression of the CRA after the suction phase is retarded compared to ideal diffusion and can be understood as a deceleration factor. In case b = −0.5, this would mean that the final d_CRA_ equals the d_CRA_ that was achieved at the end of the suction phase. A value between 0 and −0.5 results in ongoing CRA after the suction phase and during the diffusion phase, where the final re-alkalization depth is limited by the carbonation depth of the concrete but is reached after different amounts of time. In the investigation of Glawe et al., the value for b was relatively lower in the case of a > 1, leading to a stronger retardation of the CRA after the suction phase, during which a high d_CRA_ was already achieved [10]. However, the results of Glawe et al. did not reveal a clear correlation between the applied mortar and b and also assumed that variation in the progression of the CRA after the suction phase corresponded to the completed formation of the microstructure in the mortar. However, a quantification of the structural influence has not yet been undertaken.

In general, Glawe et al. mentioned further influences on the attenuation factor, namely alkali depletion in the mortar and alkali binding in the concrete [25]. The latter was primarily investigated by Réus et al., who showed clear binding of penetrating alkalis in the carbonated concrete, dependent on the concrete composition [24]. However, for these investigations, an alkaline solution was used instead of mortar as the re-alkalization material.

In the present study, the concrete substrate was intentionally kept constant in order to isolate the influence of repair mortar properties on the re-alkalization process. While the role of concrete composition on alkali binding has been addressed in previous studies, the present work focuses on mortar-related contributions to the attenuation of alkali transport [24].

This study is not intended to evaluate the overall performance of a specific repair system, but instead to identify material-related, re-alkalization-relevant properties. For this, the adapted diffusion model is not further developed in this study but instead used as a fixed analytical framework to relate material properties of the mortar and the mortar–concrete composite system to the suction factor a and the deceleration factor b. In addition to measuring the changes in the pH and electrical conductivity of the pore solution, the structural influences of the binders in the form of the formation factor and the porosity of the repair mortar on the re-alkalization process are investigated to determine their influences on the suction and deceleration factors. Furthermore, data from previously published studies on alkali concentration in HAAB-based mortars and substrate concrete during CRA are re-evaluated and discussed to complement the experimental findings of this work. By quantitatively linking mortar characteristics with the process-defining parameters a and b, the present study provides a basis for the targeted design of repair mortars for CRA.

## 2. Materials and Methods

The investigated materials are based on previous investigations by Glawe et al. in order to enable correlation of the present study and existing data [10,11]. Therefore, the used materials consisted of five mixtures of HAABs and one reference mixture without additional alkali activation. For alkali activation, a 50% potassium hydroxide solution (KOH) and a potassium water glass (KWG) (silicate modulus = 1.0) were used.

All mixtures contained 50 wt% CEM I 52.5 R and 50 wt% metakaolin, for which oxide compositions are given in Table 1.

**Figure 2:**
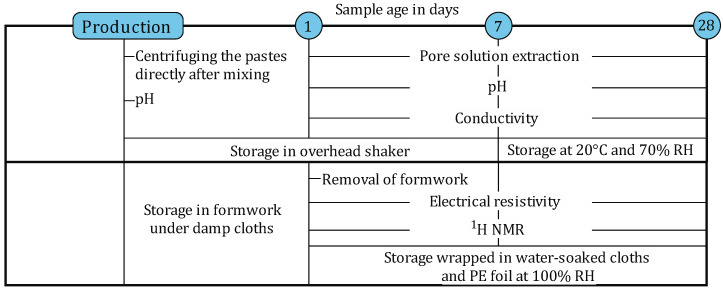
Overview of the experimental procedure applied on the mortar samples and the pore solution obtained by pore solution expression (PSE).

Both paste and mortar samples were prepared for the investigations. The paste samples were used to obtain the pore solution of the mixtures by extraction for the determination of the pH value and the electrical conductivity while the mortar samples were investigated regarding the pore structure as well as the electrical resistivity. The testing procedure is shown in Figure 2.

The compositions of the paste and mortar samples are given in Table 2 and Table 3. To ensure comparability, the w/b of 0.58 was the same for all mixtures. The activator solutions were considered in the w/b ratio in such a way that the dissolved solids, such as KOH and SiO_2_, were included in the binder component, while the water for preparing the solution was included in the liquid components.

For simplicity, the names of the mixtures were adopted from Glawe et al., where 0.5 and 2.3 represent the potassium concentration in the fresh mortar. Although this is not the case for the paste samples, the consistent names allow for better comparability of the results.

The paste samples were produced by first homogenizing the dry components and the wet components separately and then mixing all components together by stirring for 2 min.

To determine the pH value of the mixture directly after the mixing process, the fresh mixture was filled into plastic centrifuge tubes and centrifuged for 5 min at 2000 rpm. After the centrifugation, the settled solution was transferred into a 50 mL plastic bottle flooded with argon to prevent any carbonation of the solution until the time of measurement, which was a few minutes after sample production.

For the analyses of the pore solutions at further time points, the pastes were transferred into 250 mL plastic bottles flooded with argon. For each mixture, 3 bottles were filled with the paste for investigations 1, 7 and 28 days after production. The bottles were sealed by placing PE foil over the bottle opening before screwing the lid. To prevent any sedimentation during the hardening of the pastes, the bottles were placed in an overhead shaker and were shaken at 5.5 rpm. The bottles containing the samples for the investigations after 1 day were shaken for 24 h, while the samples for the 7- and 28-day measurements were shaken for 7 days. For the samples measured after 28 days, the bottles were stored at 20 °C and 70% RH.

The structural investigation was conducted using mortar samples. The components were mixed in the same way as during the production of the pastes. After mixing, the fresh mortar was transferred into a disk-shaped formwork with a diameter of 100 mm and a height of 20 mm. As the investigation of the pore structure as well as the resistivity measurements were non-destructive procedures, for the three measuring times of 1, 7 and 28 days of sample age, only one sample set consisting of three samples was produced for each mixture.

After transferring the fresh mortar into the formwork, the samples were sealed with damp cloths and PE foil to prevent the samples from drying out.

The mortar disks were taken out of the formwork after 24 h, wrapped in damp cloths and PE foil and were additionally stored at 100% RH until the measurements to ensure water saturation.

### 2.1. Extraction of the Pore Solution

**Figure 3:**
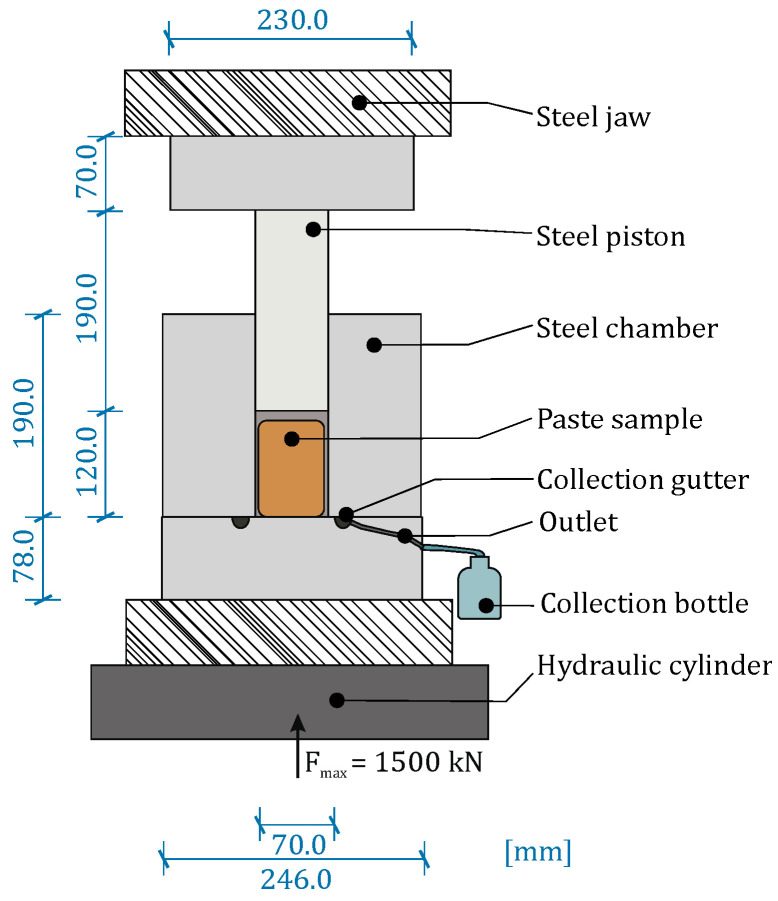
Schematic representation of the pore solution extraction device.

The extraction of the pore solution was conducted on the prepared paste samples after 1, 7 and 28 days. For the extraction process, the samples were cut out of the plastic bottles and were placed in an adapted compression testing machine, which enabled the collection of the squeezed pore solution. The compression device is schematically shown in Figure 3. Before each extraction process, all surfaces of the compression machine were cleaned using isopropanol and distilled water.

After placing the paste sample in the sample chamber, the pressure piston was introduced into the sample chamber from the top. The piston was moved at a loading speed of approximately 5 kN/s up to the maximum load of 1500 kN. During the compression process, the extracted pore solution was collected in an argon-filled plastic bottle, which was connected to the sample chamber. After each extraction process, the sample was measured immediately.

### 2.2. Measurements of the Extracted Pore Solutions

The centrifuged and extracted pore solutions were measured for their pH and electrical conductivity. The pH was measured using a pH meter 1140 (METTLER TOLEDO, Columbus, OH, USA). The results of the conductivity measurements were used to calculate the formation factor according to [26]. As the calculation of the formation factor was based on the ratio of the electrical resistivity of the pore solution and of the mortar disks, it presupposes a hardened mortar disk. Therefore, no measurements of resistivity in the centrifuged pore solutions were conducted, as no corresponding hardened mortar disks were available directly after mixing the mortar components.

**Figure 4:**
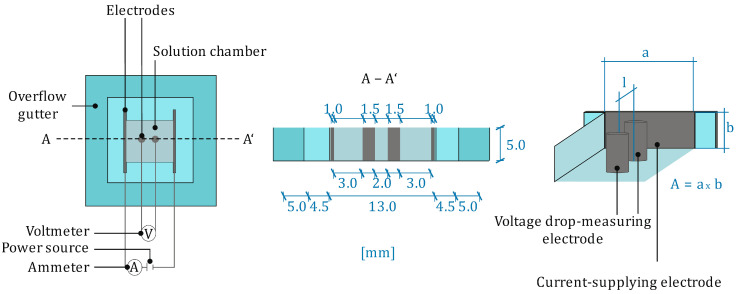
Measuring cell for the resistivity measurements of the pore solutions.

In the case of the pore solutions extracted after 1 day, electrical conductivity could not be measured using a conventional conductivity electrode. The electrode which was used in these measurements was a microprocessor conductivity meter LF 2000 (WTW). In order to still be able to determine the conductivity values, a newly developed measuring cell was used which was based on a 4-electrode method (compare Figure 4).

The measuring cell consists of two sample chambers, which are filled with the pore solution to be measured. An alternating current is introduced via the two outer electrodes, which allows the measurement of the electrical resistance between the two inner electrodes resulting from the pore solution. Following the relationship in Equation (1) to calculate the electrical resistivity,

(1) $$\rho =R*\frac{A}{l}$$

the electrical conductivity (σ) can be calculated using Equation (2) based on the measured electrical resistance (R), taking into account the geometry factor of the measuring cell (A/l).

(2) $$\sigma =\frac{1}{\rho }$$

The geometry factors for the two measuring cells are 0.0128 and 0.0131 m, respectively. For the pore solutions that could not be measured using the conductivity electrode, extracted pore solutions were measured in both cells, and the resulting conductivity was calculated by averaging the two measurements.

### 2.3. Measurements for the Mortar Disks

Mortar disks with a diameter of 100 mm and a thickness of 20 mm were measured regarding their electrical resistivity as well as their porosity in a water-saturated state. Three samples were measured for each mixture. For the resistivity measurements, a two-electrode method was used at 10,000 Hz. The coupling of the electrodes was ensured by wet cloths, which were placed between the electrodes and the sample surface.

To determine the formation factor, the measured resistivity was converted into the electrical conductivity according to Equation (2). The geometry factor was calculated by dividing the contact area, which corresponds to the cross-sectional area of the electrodes, by the height of the disk, which defines the distance between the two electrodes during the measurement.

Finally, the formation factors (FF) of the mortars were calculated using Equation (3) by dividing the electrical conductivity of the mortar, σ_Mortar_, by the electrical resistivity of the pore solution, σ_Pore Solution_.

(3) $$FF=\frac{\sigma_{Pore\;Solution}}{\sigma_{Mortar}}=\frac{\rho_{Mortar}}{\rho_{Pore\;Solution}}$$

The mortar disks were further analyzed using single-sided ^1^H NMR to obtain the porosity of the different materials at sample ages of 1, 7 and 28 days. As these measurements were conducted in the water-saturated state, we could use the same samples that we used for the measurement of the mortar resistivity.

For the NMR measurements, the NMR MOUSE PM25 (Magritek, Aachen, Germany) was used, which allows a maximum measuring depth of 25 mm at a radio frequency of 13.12 MHz.

A CPMG measurement sequence was selected, which enables data acquisition in different measuring depths. For the present study, only one measuring depth in the center of the sample at 10 mm was used to exclude any cross-sectional anomalies caused by the low thickness of the mortar disks.

At a 10 mm depth, a CPMG sequence with 1024 scans and 200 echoes, with an echo time of 0.158 ms and a recycle delay of 500 ms, was used. The pulse lengths of the 90° and 180° pulses during the measurement were 10 µs. The 180° pulse was amplified by 6 dB to maintain the size of the sensitive volume during the measurement. Using these settings, a signal-to-noise ratio (S/N) of 93.9 was achieved, which was calculated by dividing the amplitude of the first echo by the standard deviation of the last 20 echoes [27]. The resulting data are represented in the form of an exponential decay curve, which was evaluated regarding the total porosity as well as the pore types by differentiating capillary pores and gel pores within the sensitive volume. To calculate the proportion of the different pore types, the decay curve was described by the sum of two decaying exponentials using Equation (4), which were transformed into a distribution of the amplitudes (A_i_) of the different relaxation times (T_2,i_) using the inverse Laplace transformation (ILT).

(4) $$S\left(t\right)=\sum A_{i}e^{-t/T_{2,i}}$$

The resulting T_2_ distribution spectrum is related to the pore-size distribution, as the T_2_ is governed by the surface-to-volume ratio of the pores and the surface relaxivity.

The relaxation time distribution was divided into two pore domains, with a cut-off at 1 ms to distinguish between gel and capillary pores, corresponding to an often-assumed boundary between these two pore types in cementitious materials, which were used as a reference in the first approach due to the 50% cement content in the mixtures used, since no explicit values for differentiating different pore types are available for the materials at hand [27,28]. To quantify the relative pore water content as a measure of total porosity, the integrated amplitudes were normalized by the maximum amplitude of pure water, which was 3493 using the settings mentioned above [29].

### 2.4. Re-Evaluation of Previously Published LIBS Profiles of Mortar and Concrete Potassium Concentrations

To investigate the relationship between the potassium concentration of both the mortar and the concrete in the progression of CRA, the concentration gradients between the mortar and concrete were evaluated. The mean potassium concentration at the different measuring times after 1, 7 and 28 days of CRA was calculated based on the profiles measured by Glawe et al. using laser-induced breakdown spectroscopy (LIBS) [10].

**Figure 5:**
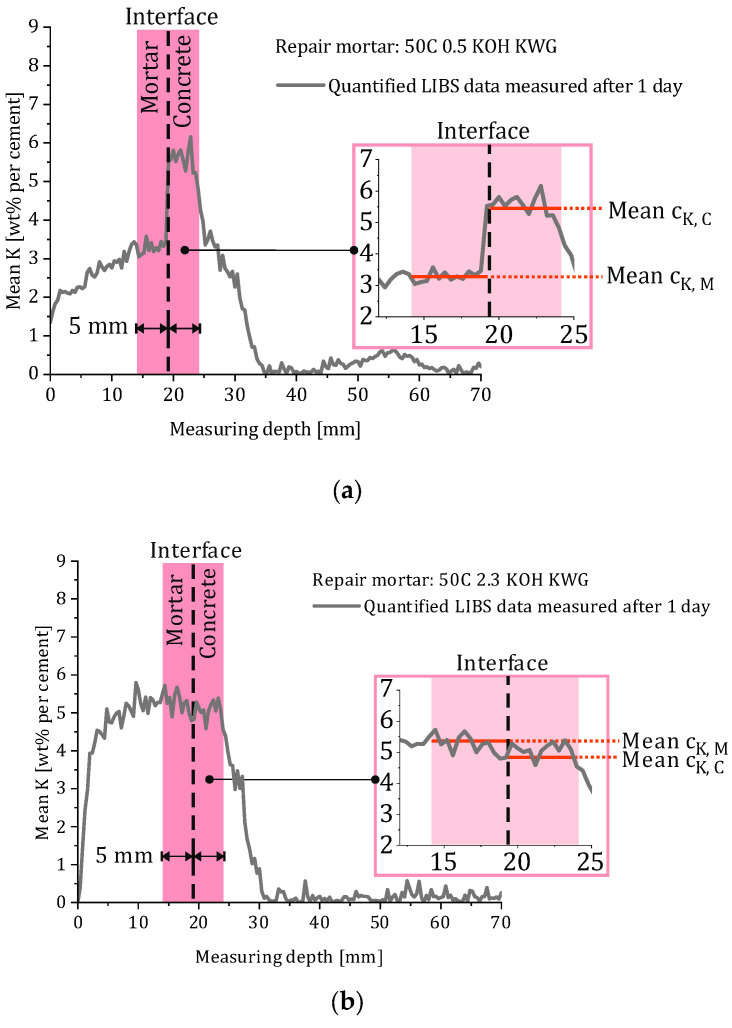
Schematic representation of the calculation of the potassium concentration at the interface area of concrete and mortar with different states of the concentration gradient, e.g., (**a**) concrete has a higher potassium concentration than mortar, (**b**) mortar has a higher potassium concentration than the concrete.

Since the potassium concentration shows a strong variation in the full cross-sectional area of the concrete and the mortar, the values were calculated based on the potassium concentration in a limited interfacial area of the composite systems to exclude external influencing factors such as leaching of the mortar. Figure 5 shows an example representation of the calculation of the mean potassium concentration of the mortar and the concrete in the interfacial area, for which a distance of 5 mm from the mortar–concrete boundary was defined as the limit.

In the investigated composite systems, two main cases regarding the concentration gradients between mortar and concrete were observed. Both cases are illustrated in Figure 5a,b). The case shown in Figure 5a involves the mortar having a higher potassium concentration than the concrete, and the other (see Figure 5b) shows a lower potassium concentration in the concrete compared to the mortar. It can already be stated at this point that the unusual phenomenon of the potassium concentration in the concrete being higher than that in the mortar is consistent with the subsequent development of re-alkalization over time, as discussed later. However, it is important to consider that the LIBS analysis method used for the determination of the potassium concentration measures a combination of potassium in the solid state and potassium dissolved in the pore solution of the material.

Using the calculated values for c_K,M_ and c_K,C_, the concentration gradient of potassium, Δc_K_(t), was calculated according to Equation (5) for the values of 1, 7 and 28 days.

(5) $${\Delta c}_{K}(t)=c_{K,M}(t)-c_{K,C}(t)$$

Based on the values for Δc_K_ of each mortar–concrete combination, the potassium concentration gradient decay rate (CGDR_K_) was calculated using Equation (6).

(6) $${CGDR}_{K}=\frac{\Delta c_{K}\left(28\;d\right)-\Delta c_{K}\left(1\;d\right)}{28-1}$$

The CGDR_K_ of the time periods of 1 to 7 and 7 to 28 days was calculated equally. The resulting values for CGDR_K_ quantify the rate at which the potassium concentrations in the mortar and the concrete equalize over the different durations of CRA of 6, 21 and 27 days.

## 3. Results

The adaption of Fick’s second law of diffusion to the progression of the CRA is based on the attenuation factor β(t), which is defined by the two factors a and b. Previous studies by Glawe et al. have shown that both factors depend on the mortar used, which is applied to the substrate concrete [10]. However, no direct correlation has yet been established between specific material properties and factors a and b.

In the following results, the material properties of mortar and concrete are systematically correlated with these two factors in order to determine their influences on CRA. The values for the factors which were determined by Glawe et al. are given in Table 4.

It should be noted in all considerations that the values were determined using laboratory samples that were not applied as a repair layer to concrete. The exception to this is the determination of the mean potassium concentration in the mortar and concrete layer, which was measured by LIBS on mortar applied to concrete.

Due to transport processes during CRA, changes in other mortar properties or pore solution properties, in addition to changes in potassium concentration, must also be assumed, which are not considered in this study.

The number of analyzed specimens (n) is given in the caption of each figure. For the correlation, error bars are not shown, as correlated parameters were derived from single-specimen measurements or mean values to indicate trends rather than provide statistically robust relationships. Unless stated otherwise, measurements of the pore solution properties (pH value and conductivity), mortar porosity and LIBS-based potassium concentration profiles were performed on a single specimen for each mortar composition. Electrical resistivity measurements of the hardened mortars were conducted on three replicate specimens, and the corresponding formation factors were calculated accordingly.

### 3.1. Chemical Characterization of the Repair Mortars

The investigation of the influence of the chemical properties of mortar on CRA was carried out using the pH value and the electrical conductivity of the pore solution.

**Figure 6:**
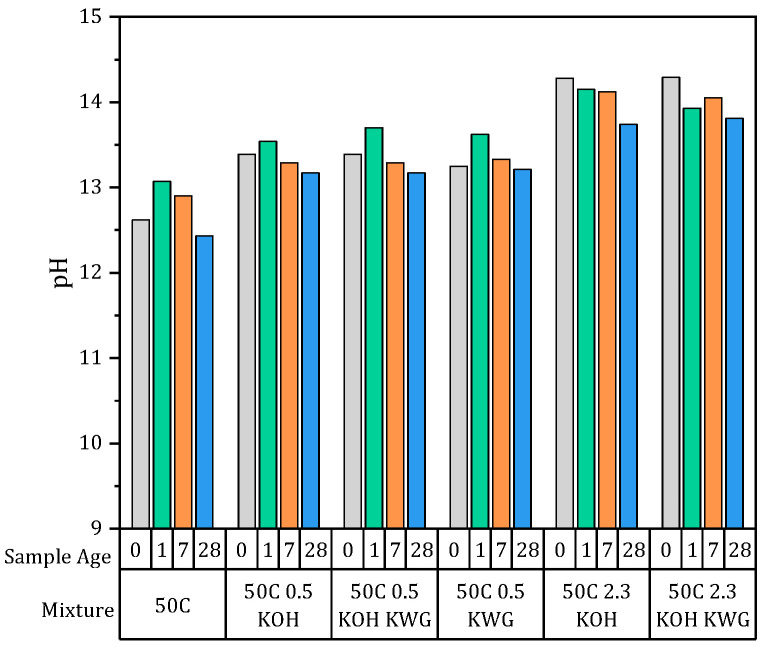
pH values of the different mixtures in the fresh state and after 1, 7 and 28 days (n = 3).

**Figure 7:**
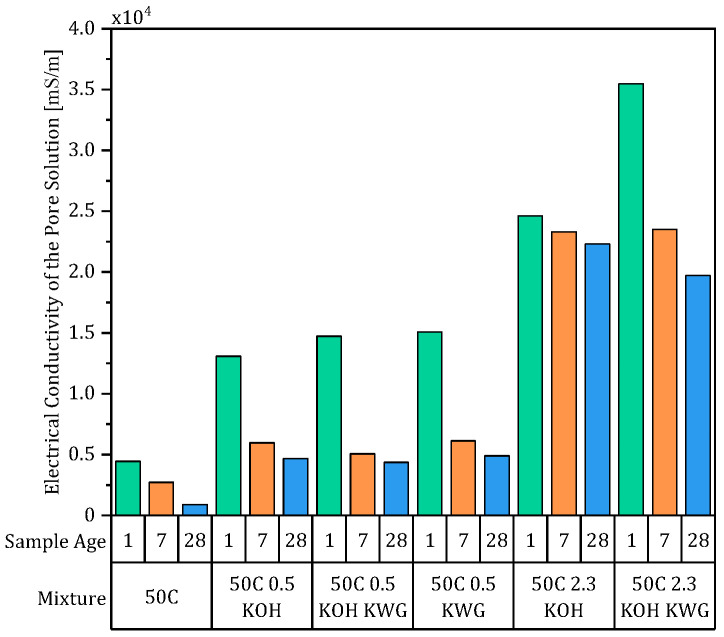
Electrical conductivity of the different mixtures after 1, 7 and 28 days (n = 1).

The temporal changes in the pH and the electrical conductivity of the pore solution of the materials are shown in Figure 6 and Figure 7. The measurements were conducted on one sample for each mixture. Regarding the changes in the pH over time, the mixtures can be divided into two groups. The non-activated 50C mixtures and the mixtures with a mortar potassium content of 0.5 wt% showed similar behaviors, as the pH increased between the fresh-state sample and the sample aged by 1 day. The second group consists of the mixtures which were alkali-activated, resulting in a potassium content of 2.3 wt%, showing the highest pH in the fresh state, which then decreased with increasing sample age. However, a higher potassium concentration resulted in a higher pH for the pore solution, giving the mixtures with 2.3 wt% the highest pH in the fresh state, with values over 14, as well as after 28 days, while the non-activated mixture showed the lowest pH values, which dropped below 12.5 after 28 days. The mixtures with the lower potassium concentration of 0.5 showed a similar behavior, although different activator solutions were used.

Since the electrical conductivity was primarily measured to calculate the formation factor, no values for the conductivity in the fresh state are available.

However, the mixtures exhibit similar behavior to the pH values, showing decreasing conductivity with increasing sample age. The grouping of the mixtures is also present here, with the mixture with high potassium concentration and alkali activation using KOH and KWG showing a very high conductivity after one day. Overall, the pH and the electrical conductivity show systematic, time-dependent changes, with decreasing values at increasing sample ages.

### 3.2. Structural Characterization of the Repair Mortars

To see how CRA is influenced by the structure of the mortars, two different approaches were pursued. First, the formation factor was calculated at sample ages of 1, 7 and 28 days. Second, and at the same sample ages, the porosity was measured using single-sided ^1^H NMR.

**Figure 8:**
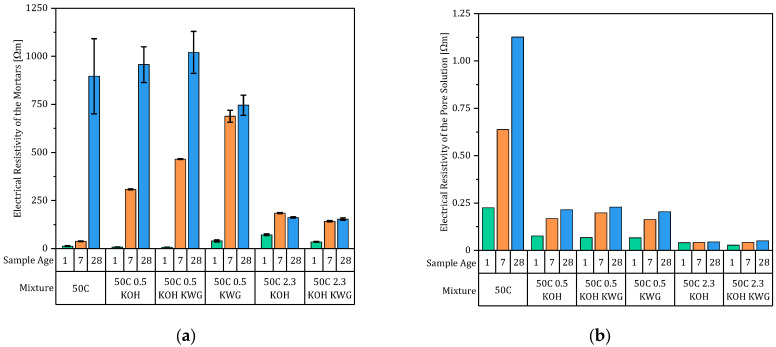
Electrical resistivities (n = 3) of the mortars (**a**) and the corresponding pore solutions (**b**) (n = 1).

**Figure 9:**
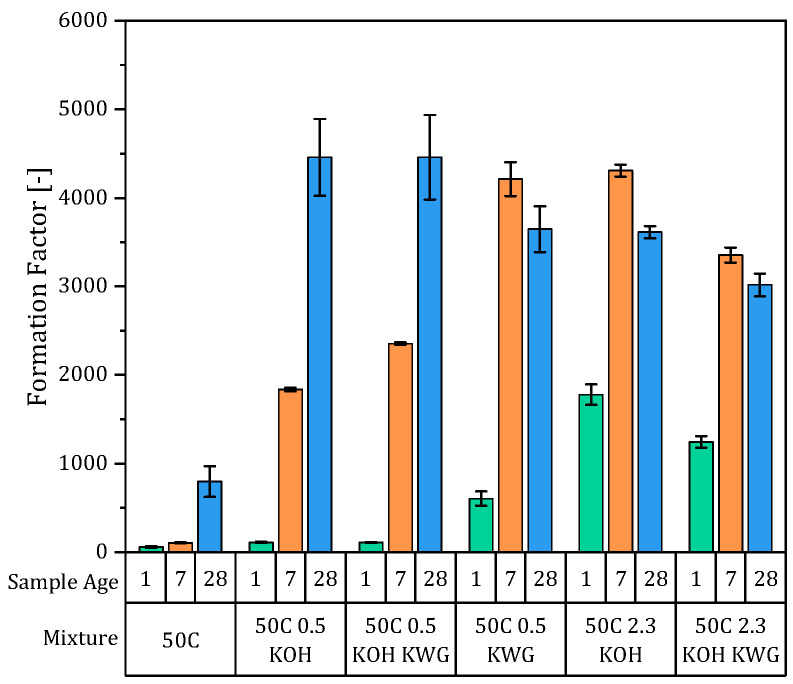
Formation factors of the different mortars at sample ages of 1, 7 and 28 days.

The electrical resistivity of the mortars, which results from the average of three individually measured samples, and the pore solutions are given in Figure 8a,b, and the resulting formation factors, which were calculated according to Equation (3), are shown in Figure 9 for each mixture at the different sample ages. Again, the mixtures can be divided into two groups based on the deviating temporal changes in the formation factor with increasing sample age. The non-alkali-activated mixture and the alkali-activated mixtures with low potassium content both demonstrated, regarding their pore solution and mortar state, an increasing electrical resistivity and a resulting increasing formation factor with increasing sample age. However, an exception is the mixture that was activated exclusively with KWG, which showed a decreasing formation factor between 7 and 28 days. Looking at the resistivity of the pore solution and of the mortar of this mixture separately, the mortar resistance increased by less between 7 and 28 days compared to the other two mixtures with low potassium concentrations. This resulted in a nearly constant resistance at both 7 and 28 days for this mixture. In contrast, the development of the pore solution resistance in the KWG-activated mixture corresponded to those of the other mixtures with low potassium concentrations over time.

The second group based on the formation factor consists of mixtures with high potassium content that first show an increasing trend up to a sample age of 7 days and then a decreasing trend between 7 and 28 days. This is the result of very low mortar resistances at high potassium concentrations, which, unlike the other mixtures, cause them to show a constant or decreasing resistivity. Since the samples were wrapped in water-soaked cloths for storage prior to measurement, leaching in the samples was to be expected, which was assumed to hinder the formation of the material’s structure. This was confirmed by the constant electrical resistance of the mortar disks after 7 and 28 days.

**Figure 10:**
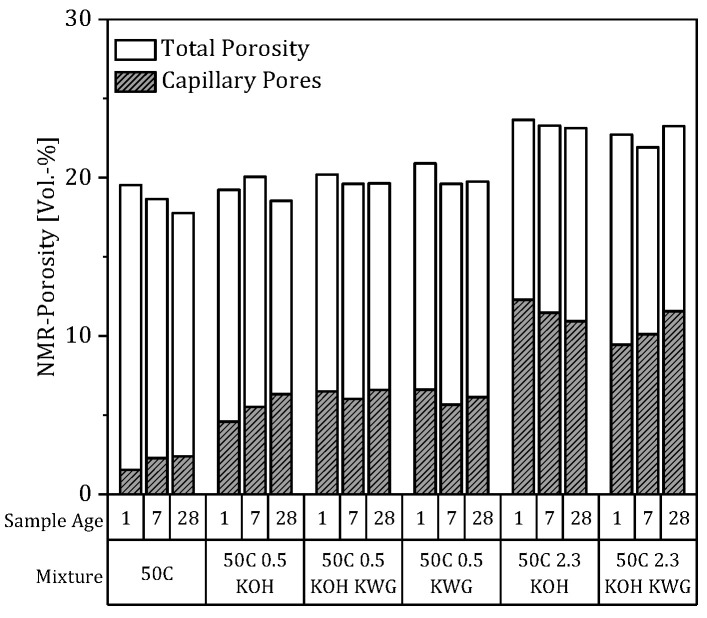
Total porosity and proportion of capillary pores of the mortar mixtures (n = 1).

Since the formation factor indicates the structural influence on the conductivity of the mortar and results from the porosity and connectivity of the pores, the porosity of the mixtures was also determined (compare Figure 10).

Within the NMR porosity analysis, only slight differences in total porosity are discernible between the various mixtures. In contrast, the evaluation of the capillary pore fraction shows strong differences, and three fractions can be distinguished.

The first fraction consists of the non-activated mixture, 50C, showing a decreasing total porosity over time from 19.5 to 17.8 Vol.-%, while the capillary pores increase but are almost constant between 7 and 28 days. This already shows the weak correlation between the NMR porosity and the formation factor, which shows stronger temporal changes than the results of the NMR measurements regarding all mixtures. Particularly interesting is the fact that this mixture already exhibits a well-developed porosity with a low proportion of capillary pores after just one day, indicating a dense structure. In contrast, the formation factor is low at sample ages of 1 and 7 days, meaning the developed structure had only a minor influence on the electrical resistance of the mortar, leaving pore connectivity as the main defining parameter.

The second fraction consists of the alkali-activated mixtures with low potassium concentrations that showed an overall similar total porosity as well as a capillary porosity between 4.6 and 6.6 Vol.-%. The third fraction is defined by a higher total porosity of between 21.9 and 23.6 Vol.-% in the case of mixtures with potassium concentrations comparable to the other fraction. These mixtures also show the highest proportion of capillary pores, which decreases over time for the KOH-activated mixture and increases over time in the case of the KOH-KWG-activated mixture.

Although temporal trends in porosity within a mortar mix were evaluated in some cases, these were very small and showed no systematic correlation with the course of the CRA. Due to the small magnitudes of the observed changes and the associated uncertainty of the porosity measurements, no further interpretation of the results was undertaken. The results suggest that potential microstructural changes remain secondary compared to chemical effects under the investigated conditions.

However, alkali activation and potassium concentration in the mixtures had an influence on the total porosity, as well as the pore-size distribution represented by the capillary pores.

### 3.3. Concentration Gradient Decay Rate

**Figure 11:**
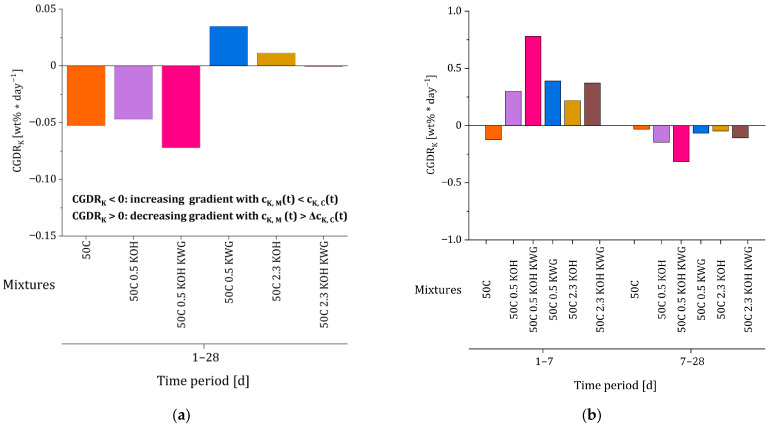
Results of the potassium concentration gradient decay rate (CGDR_K_) calculated based on the LIBS measurements of Glawe et al. [10] for the different time periods: (**a**) 1 to 28 days, (**b**) 1 to 7 and 7 to 28 days (n = 1).

The calculated potassium concentration gradient decay rates (CGDR_K_), which are based on the concentration gradients given in Table 5, are presented in Figure 11a,b. The LIBS-based concentration profiles were based on a single specimen per mortar composition (n = 1). Mean concentration profiles were used for further evaluation in order to reduce the influence of local measurement noise. Due to the pronounced signal scatter inherent to the LIBS technique, no standard deviations are displayed for the derived concentration gradients. Since the values will later be correlated with the deceleration factor b, which was calculated based on a re-alkalization period of 28 days, the following section focuses on the calculated decay rate values for the period between 1 and 28 days (compare Figure 11a). However, the decay rates for the periods between 1 and 7 days and 7 and 28 days were also calculated (compare Figure 11b).

Based on the results for the period of 1 to 28 days, the investigated composite systems can be divided into two groups. The systems with CGDR_K_ < 0 are characterized by an increasing potassium concentration gradient over time between the mortar and the substrate concrete, with a potassium concentration in the concrete which exceeds the potassium concentration in the mortar. This is present when applying mortars which are alkali-activated using KOH or a mixture of KOH and KWG with a potassium concentration of 0.5 wt% or when applying a non-activated mortar with a substitution rate of 50%. Such a gradient results in a decelerating potassium transport from the mortar into the substrate concrete.

The second group with CGDRK > 0 or nearly CGDRK = 0 includes mortars with high potassium concentrations, as well as the mortar with a low potassium concentration which was alkali-activated using only KWG. These composite systems are characterized by a constant or decreasing potassium concentration gradient between the mortar and concrete, with a permanently higher potassium concentration in the mortar than in the concrete, resulting in ongoing potassium transport from the mortar into the substrate concrete.

### 3.4. Influences on Early-State CRA

In general, CRA is divided into two phases, the suction phase, which takes place during the first 24 h after the repair mortar is applied, and the diffusion phase, which encompasses all points in time after the suction phase.

The early state of CRA during the suction phase is characterized by a high re-alkalization rate, which has been assumed to be mainly driven by the moisture gradient between the mortar and the substrate concrete until now [19]. Additionally, Glawe et al. observed a clear positive correlation between the re-alkalization rate during the suction phase and the alkali concentration in the mortar [10]. It was further stated that the transport processes during the suction phase and the diffusion phase can be mathematically described by an adaption of Fick’s second law of diffusion using the attenuation factor β(t). Including the attenuation factor, which is defined by the two factors a and b, the early state of CRA is adapted by the factor a.

**Figure 12:**
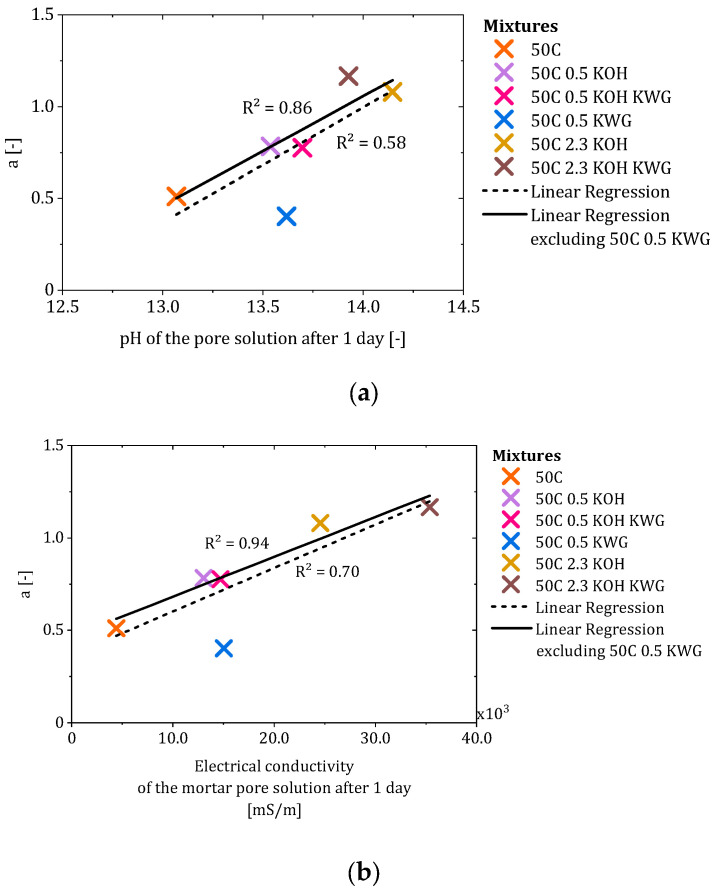
Correlation of the pH (**a**) and the electrical conductivity (**b**) of the pore solution of the mortar after 1 day with the suction factor a.

Since a correlation between the potassium concentration of the applied mortar and the re-alkalization rate during the suction phase was already observed by Glawe et al., the alkali concentration in the mortar was analyzed based on the pH and the electrical conductivity of the pore solution of the mortar in the present work. The correlation of the pH and the electrical conductivity of the pore solution of the mortar after one day with the factor a is shown in Figure 12a,b.

The data were correlated using a linear regression to calculate the coefficient of determination to compare the correlations of the different properties with the factor a. The parameter a represents the early-stage progression of chemical re-alkalization during the suction phase and is commonly interpreted as a measure of the initial transport behavior, which is based on varying moisture and concentration gradients between mortar and concrete based on the applied mortar. For the chemical properties of the pore solution of the mortar, a correlation between the pH and the electrical conductivity of the pore solution can be observed. The coefficient of determination increases after the exclusion of the mortar 50C 0.5 KWG, which showed a high pH and electrical conductivity and a comparably low value for factor a, which led to a low re-alkalization rate during the suction phase compared to other mixtures with similar pore solution properties. However, the pore solution was shown to have a strong influence on factor a and therefore the re-alkalization depth that can be achieved during the suction phase. This interpretation is consistent with the adapted description of the CRA process by Glawe et al., where factor a primarily reflects mortar-related influences during the initial suction phase. A high initial pH for the pore solution results in a high concentration gradient between the mortar and concrete, which leads to increased transport from a high to low concentration by piggyback convection in order to achieve concentration equilibrium. Such an influence can also be assumed with regard to the moisture gradient, which is probably highest at the beginning of the suction phase, but this could not be proven based on the available data.

**Figure 13:**
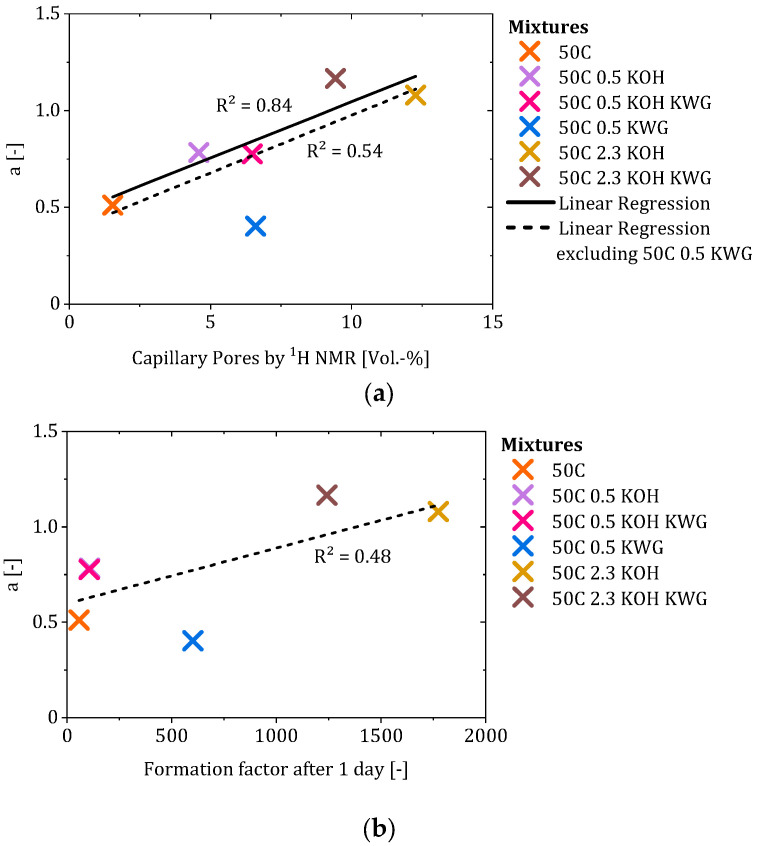
Correlation of the capillary pores (**a**) and the formation factor (**b**) of the mortars with the suction factor a.

Since the investigated mortars showed variation regarding structural properties such as the formation factor, an influence of these properties in the early state of CRA could be assumed. Therefore, the formation factor and the capillary porosity of the mortars were correlated with factor a (compare Figure 13a,b).

The correlation of the capillary pores and factor a showed a coefficient of determination of 0.54, which increased to 0.84 when the mortar 50C KWG was excluded. Since the chemical properties also showed a deviation, excluding this mixture seemed sensible in order to check the relationships between the properties of the other mortars and factor a. As a result, the proportion of capillary pores in the mortar appeared to influence the progression of CRA during the suction phase. Based on the literature, the water present in capillary pores is often characterized as mobile water which can move through the pore structure of the material [28]. This leads to the assumption that the achieved re-alkalization depth during the suction phase depends on the mobile water present in the mortar, which acts as a transport medium for the alkalis in the form of piggyback transport [30].

In the case of the correlation of the formation factor after 1 day, only a weak correlation (R^2^ = 0.48) could be observed, which made the exclusion of the 50C KWG mixture seem unnecessary. However, the results would mean that a higher formation factor would lead to a higher re-alkalization rate during the suction phase. As the formation factor characterizes the structural influence on the conductivity of the mortar in terms of ion transport through the pore system of the material, the present weak correlation is contradictory [26]. Therefore, the chemical composition of the pore solution of the mortar is the driving force during early-state CRA, while the investigated structural properties of the mortar seem to play only a subordinate role.

### 3.5. Influences on Later-State CRA

The progression of CRA after the suction phase is defined by the deceleration of the process which is given by the factor b. Based on previous research by Glawe et al., factor b cannot be explained simply by the initial characteristics of the applied mortar, e.g., the potassium concentration [10]. Rather, it seems likely that changes in the mortar or concrete over time have a direct influence on the deceleration of the CRA during the diffusion phase. In an initial approach, Glawe et al. summarized this through the structural development and decreasing alkali buffer of the mortar, as well as the alkali binding in the concrete [10].

**Figure 14:**
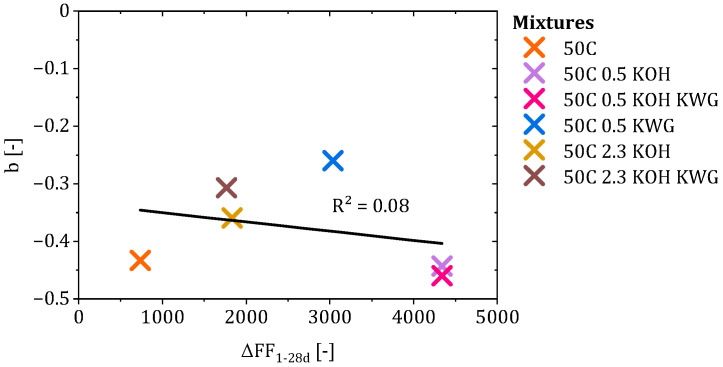
Correlation between the deceleration factor b and the changes in the formation factor of the mortars at sample ages of 1 and 28 days.

Therefore, structural changes in the mortar in the form of the difference between the formation factors at 1 and 28 days were correlated with factor b. The results of the correlation are given in Figure 14 and show that there is no connection between the structural changes in the mortar and the decelerating behavior of CRA.

A correlation between structural changes in the form of porosity, determined using ^1^H NMR, is omitted here, as the mortars do not show any significant changes over time based on the available results.

**Figure 15:**
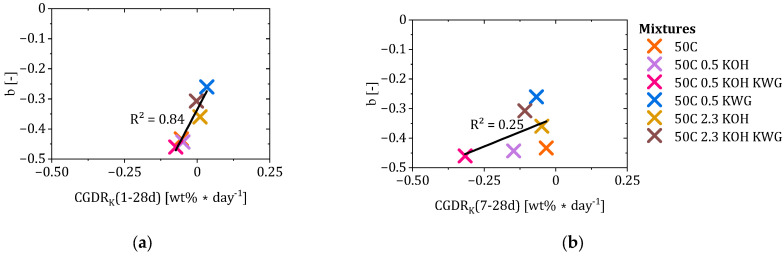
Correlation of the deceleration factor b and the potassium concentration gradient decay rate between 1 and 28 (**a**) and 7 and 28 (**b**) days of CRA.

To include the interaction of the alkali concentration in the mortar and the concrete, the CGDR_K_ values of different time periods were calculated and also correlated with b, as it describes changes in potassium concentration differences in the concrete and the mortar in the interfacial area of the composite system. Since the deceleration factor b defines the progression of CRA in the later state, only a correlation was conducted with the values for CGDR_K_ of the periods between 1 and 28 and 7 to 28 days (compare Figure 15a,b). The results showed a high apparent correlation between the changes in the potassium concentration gradient during the re-alkalization period from 1 to 28 days and the deceleration factor b (r = 0.95 with *p* = 0.004; ρ = 0.89 with *p* = 0.002). Despite the limited number of mortar mixes (n = 6), the low *p*-values indicate that the observed correlation was unlikely to be purely random; however, the wide confidence interval (95%-Cl: [0.58; 0.99]) reflects considerable uncertainty, and the correlation should therefore be interpreted as indicative rather than statistically robust.

In the case of values for CGDR_K_ < 0, which indicates a decreasing gradient between the mortar and concrete, leading to a potassium concentration in the concrete that equals or exceeds the potassium concentration of the mortar, the value of b becomes more negative. As b approaches the value −0.5, the maximum achievable re-alkalization depth is increasingly dominated by the suction phase. Due to the relationship between b and CGDR_K_, a rapidly decreasing concentration gradient between the mortar and concrete in the interfacial area leads to a significant deceleration of the CRA during the diffusion phase.

Regarding the investigated mortars, the concentration gradient shows the highest decay rate for mortars with low potassium concentrations. The exception is the mortar 50C 0.5 KWG, which shows a CGDR_K_ > 0 and the highest value for b, which means that the CRA shows a strong progression during the diffusion phase. However, this mortar only shows a final re-alkalization depth that is lower compared to the mortars with high potassium concentrations, which is based on the different behaviors of the mortars during the suction phase. It has to be noted that, despite the deviating behavior of the mixture 50C 0.5 KWG, exclusion of this mixture did not need to occur to obtain a high coefficient of determination.

The mortars with a high potassium concentration were characterized by a CGDR_K_ of approximately 0, meaning that the concentration gradient between the mortar and concrete remained nearly constant over the investigated re-alkalization period of 28 days. In combination with a high re-alkalization rate during the suction rate, these mortars showed the highest final re-alkalization depths [10].

In comparison, only a weak correlation of the deceleration factor b and the values for CGDR_K_ between 7 and 28 days could be observed. This confirms that the deceleration factor is sensitive to changes in the concentration gradient between the mortar and concrete throughout the entire diffusion phase. Therefore, a comparison with CGDR_K_ values at 1 to 7 days would be misleading, as these values also reflect processes from the initial suction phase.

## 4. Discussion

The present study focuses on the determination of the influences of HAAB-based mortars and concrete on the progression of CRA during different phases of the re-alkalization process. For this purpose, various properties of mortar and concrete were correlated with the factors a and b, which were taken from a previous study by Glawe et al., as these characterize the course of CRA during the initial suction phase and the subsequent diffusion phase, respectively [10].

A direct quantitative comparison with previous, independent studies is limited, as HAABs have so far not been systematically investigated as repair mortars for CRA by other researchers. Existing studies primarily focus on OPC-based repair mortars or alkaline solutions, which strongly differ in terms of pore solution chemistry, hardening processes and transport mechanisms. This is mainly due to the fact that OPC-based mortars have so far only been investigated at low carbonation depths of up to 14 mm, and alkaline solutions differ significantly from the properties of HAAB-based mortars due to a permanently, significantly increased moisture supply [17,19]. However, across all studies, a correlation is evident with regard to the differentiation of the individual phases of CRA for all the repair materials used.

In the beginning of CRA, the suction phase starts directly after the repair mortar is applied. During this phase, it becomes apparent that the alkalinity of the mortar, in the form of the pH of the pore solution and the electrical conductivity, and the available mobile water, in the form of the capillary pores, have a significant influence on the course of the CRA. Especially at very high pH values (pH > 14), small changes in pH correspond to large changes in hydroxide ion concentration due to the logarithmic nature of the pH scale. Consequently, mortars exhibiting pH values above 14 possess a substantially larger alkali depot, which can sustain strong alkali emission in the early state without rapid depletion.

In comparison to existing studies by Reus et al., the use of a pure KOH solution results in an even more pronounced suction phase. Although the studies do not provide re-alkalization values after 1 day, almost complete re-alkalization was achieved after just 7 days, whereas with the use of HAABs, approximately 50% of the maximum re-alkalization depth was reached within this time period [31]. Accordingly, it can be assumed that, under otherwise identical conditions, the value for the absorption factor a would exceed those of HAAB-based mortars when using alkaline solutions. The investigations by Merkel would accordingly show a significantly reduced absorption factor (a < 1), since cement-based mortars exhibit a less pronounced absorption phase, which can still be differentiated from the subsequent diffusion phase [19]. The results of Bier et al. also confirm the presence of a less pronounced suction phase when using cement-based repair mortars, even though such a distinction was not explicitly made in these studies [17,32].

Although the proportion of capillary pores represents a structural parameter of the mortars and provides pathways for ionic transport, the proportion alone does not determine the re-alkalization rate during the suction phase. The formation factor, as an additional structural parameter derived from the electrical resistivity of the pore solution and the mortar, shows a non-systematic relationship with the re-alkalization rate during the suction phase. This illustrates that, while the capillary pores are a prerequisite for moisture-based transport, their proportion primarily reflects the amount of mobile pore water instead of the transport kinetics. A similar proportion of mobile water does not necessarily lead to a similar value for the suction factor a, as is the case for the mixtures of 50C 0.5 KOH KWG and 50C 0.5 KWG. Here, 50C 0.5 KWG shows a much lower value for factor a that results in relatively lower CRA during the suction phase compared to 50C 0.5 KOH KWG, while both mixtures show similar capillary pores. This underlines that, overall, the early-stage progression of CRA is governed predominantly by the chemical composition of the pore solution, which can outweigh purely structural effects under highly alkaline conditions and under the investigated conditions [31].

The diffusion phase starts after the suction phase, which is assumed to take a maximum of 24 h. In previous research, the transition from the suction phase to the diffusion phase has been characterized by a deceleration of the process [11,33]. This deceleration was introduced as the factor b by Glawe et al. and enabled adaptation of Fick’s second law of diffusion to the re-alkalization process [10].

To determine the influences on the deceleration behavior of CRA during the diffusion phase, the changes in mortar and concrete properties over time were correlated with factor b. This again showed that the developing structural influence of the mortar, in the form of the formation factor, has no correlation with b. Since the proportion of the capillary pores in the mortars shows only minor changes over time, no systematic relationship between the investigated structural parameters and the re-alkalization behavior during the diffusion phase could be identified within the present dataset.

In contrast to this, the potassium concentration gradient decay rate CGDRK shows a very strong correlation with the deceleration factor b, leading to the conclusion that the concentration gradient at the mortar concrete interface dominates the progression of the CRA during the diffusion phase. According to this, the strongest deceleration in the form of relatively more negative values for b can be observed for the mortars with the highest CGDR_K_ between mortar and concrete. In the case of the mixtures 50C 0.5 KOH and 50 C 0.5 KOH KWG, the potassium concentration of the concrete exceeds the concentration equilibrium between mortar and concrete, which indicates binding of potassium in the near interfacial area of the concrete [24]. Therefore, factor b is defined by a combination of mortar and concrete properties. More precisely, factor b indicates how many alkalis the mortar can provide and how quickly, in relation to the absorption rate and absorption capacity of the concrete. In the case of the mixture 50C 0.5 KWG, which is characterized by a low factor a and a less negative value for factor b, the mortar releases the alkalis more slowly but over a longer period of time compared to the other mixtures with similar potassium concentrations, resulting in a lower CGDR_K_.

A comparison with existing studies by Merkel and Reus, which used cement-based mortars or alkaline solutions for re-alkalization, respectively, confirms the interpretation of the decelerating factor b [19,31]. In the case of cement-based mortars, which typically have an initial pH of 12.5–13.5, only a weakly pronounced suction phase is observed, followed by a diffusion phase with minimal deceleration. Due to the low release during the absorption phase, the CRA is only weakly decelerated during the diffusion phase. In the case of the alkaline KOH solution, which is characterized by a very high, easily available alkali depot, the diffusion phase is only weakly decelerated even after a strong suction phase. Although no explicit pH values for the solution are given, a pH of approximately 14.4 can be assumed for the described solution with 150 g/L KOH [31].

It is important, however, not to evaluate the potassium concentration of the entire mortar layer or the bulk concrete, as leaching can occur in the near-surface area of the mortar as a result of cyclic irrigation, which would distort the average values for the potassium concentration. However, in the present study, cyclic irrigation results in continuous but controlled leaching under identical exposure conditions for all composite systems. Such leaching effects were already observed by Glawe et al. and occurred primarily in mortars with high potassium concentrations, which nevertheless exhibited only a weak slowdown during the suction phase [10]. This indicates that, under the investigated conditions, leaching affects local concentration levels but does not dominate the overall progression of CRA. However, under scenarios involving permanent water contact, such as applications in drinking-water environments, more pronounced leaching may occur and should be considered separately.

## 5. Conclusions

The investigations presented here provide the first quantitative, parameter-based evaluation of the material-related influences of HAAB-based repair mortars on CRA. Based on the systematic correlation of the obtained results for the investigated materials with the existing adapted diffusion model, the following conclusions can be drawn.

- Using the adapted diffusion framework, this study provides the first quantitative correlation of mortar- and concrete-related properties and the re-alkalization-defining parameters, the suction factor a and the deceleration factor b.
- The suction factor a defines CRA during the initial suction phase and is mainly influenced by the alkalinity of the mortar pore solution, with pH values exceeding 14 leading to pronounced early-stage CRA with a > 1.
- Strong re-alkalization (a > 1) during the suction phase does not inevitably lead to a strong deceleration of CRA, as the potassium concentration gradient between the mortar and concrete is maintained in the case of initial mortar pore solution pH values above 14, as such high pH values correspond to substantially higher alkali concentration.
- The influences on the diffusion phase, which are defined by the deceleration factor b are dominated by the concentration gradient decay rate CGDR_K_, which describes the temporal development of the potassium concentration gradient between the mortar and concrete in the interfacial area during the diffusion phase.
- The structural properties of the applied repair mortar seem to have a less pronounced influence on both the suction factor a and the decelerating factor b, suggesting that future optimization of repair mortars should primarily address pore solution chemistry rather than pore structure alone.

## 6. Outlook

The present investigations focused primarily on the mechanisms of chemical re-alkalization and the influence of mortars and the substrate concrete on this process. While the presented influences of the investigated materials show good correlation results with the introduced factors a and b, further material variations should be analyzed to verify the results of this study. Furthermore, additional substrate concretes with varying structural properties and binders should be investigated, as there has been only one type of concrete used so far. Since one of the mortar mixtures shows deviating behavior, the mortar composition should also be varied in regard to binder and activator solutions in future research. In addition, previously used clinker-reduced binders could also be tested as repair mortars.

## Figures and Tables

**Table 1:** Oxide composition of the used materials.

Component	Unit	CEM I 52.5 R	Metakaolin
SiO_2_	wt%	20.21	60.97
Al_2_O_3_		5.61	35.01
Fe_2_O_3_		2.15	1.26
MnO		0.06	0.00
TiO_2_		0.33	1.63
P_2_O_5_		0.28	0.04
CaO		64.46	0.24
MgO		1.57	0.13
K_2_O		1.00	0.51
Na_2_O		0.11	0.06
SO_3_		3.85	0.04
Sum		99.63	99.92
LOI		2.60	1.03

**Table 2:** Mixtures of the investigated pastes based on [10].

		50C	50C 0.5 KOH	50C 0.5 KOH-KWG	50C 0.5 KWG	50C 2.3 KOH	50C 2.3 KOH-KWG
Cement Type	-	CEM I 52.5 R					
Cement	kg/m^3^	534.7	522.3	520.6	518.9	467.9	459.5
Metakaolin		534.7	522.3	520.6	518.9	467.9	459.5
Water		624.6	599.2	596.5	593.8	488.1	475.9
KOH_sol_ (50%)		-	52.2	26.0	-	280.7	137.9
Potassium Waterglass (silicate modulus = 1.0)		-	-	34.7	69.1	-	183.7
w/b	0.58						
Potassium in fresh paste (calculated)	wt%	0.40	1.46	1.45	1.44	6.08	5.93

**Table 3:** Mixtures of the investigated mortars based on [10].

		50C	50C 0.5 KOH	50C 0.5 KOH-KWG	50C 0.5 KWG	50C 2.3 KOH	50C 2.3 KOH-KWG
Cement Type	-	CEM I 52.5 R					
Cement	kg/m^3^	225.3	223.0	222.7	222.4	212.4	210.7
Metakaolin		225.3	223.0	222.7	222.4	212.4	210.7
Aggregates							
0.0–0.25 (quartz powder)		355.5	341.2	340.7	340.3	324.9	322.3
0.1–2.0		996.1	996.8	995.5	994.1	949.2	941.7
Water		263.1	255.8	255.2	254.5	221.5	218.2
KOH_sol_ (50%)		-	22.3	11.1	-	127.4	63.2
Potassium Waterglass (silicate modulus = 1.0)		-	-	14.8	29.6	-	84.2
w/b	0.58						
Potassium in fresh mortar (calculated)	wt%	0.14	0.50	0.50	0.50	2.3	2.3

**Table 4:** Values for the factors a and b taken from [10].

	50C	50C 0.5 KOH	50C 0.5 KOH KWG	50C 0.5 KWG	50C 2.3 KOH	50C 2.3 KOH KWG
a	0.507	0.781	0.773	0.398	1.078	1.165
b	−0.435	−0.445	−0.462	−0.261	−0.361	−0.308

**Table 5:** Results of the calculation of the potassium gradient between mortar and concrete after 1, 7 and 28 days according to Equation (5) (n = 1).

	50C	50C 0.5 KOH	50C 0.5 KOH KWG	50C 0.5 KWG	50C 2.3 KOH	50C 2.3 KOH KWG
Δc_K_(1)	0.081	−0.409	−1.019	−1.291	−0.935	−1.319
Δc_K_(7)	0.070	1.393	3.663	1.048	0.353	0.912
Δc_K_(28)	−0.609	−1.679	−2.968	−0.353	−0.648	−1.337

## Data Availability

The original contributions presented in this study are included in the article. Further inquiries can be directed to the corresponding author.

## Abbreviations

The following abbreviations are used in this manuscript:

CRA	Chemical re-alkalization
CGDR_K_	Potassium concentration gradient decay rate
Δc_K_(t)	Difference in the potassium concentration between mortar and concrete at a specific time point
D_K,1d_(RM)	Specific potassium diffusion coefficient of the concrete dependent on the repair mortar measured after one day
FF	Formation factor
HAAB	Hybrid alkali-activated binder
KWG	Potassium water glass
LIBS	Laser-induced breakdown spectroscopy
OPC	Ordinary Portland cement
